# Physical and psychological correlates of somatic symptom in patients with functional constipation: a cross-sectional study

**DOI:** 10.1186/s12888-024-05559-9

**Published:** 2024-02-16

**Authors:** Zhifeng Zhao, Bin Bai, Shiqi Wang, Yin Zhou, Pengfei Yu, Qingchuan Zhao, Bin Yang

**Affiliations:** State Key Laboratory of Cancer Biology & National Clinical Research Center for Digestive Diseases, Xijing Hospital of Digestive Diseases, Air Force Medical University, No.127, West Changle Road, 710032 Xi’an, Shaanxi Province People’s Republic of China

**Keywords:** Somatic symptom, Functional constipation (FC), Anxiety, Depression, Patient Health Questionnaire, Cross-sectional study

## Abstract

**Background:**

The symptoms of functional constipation (FC) were obviously affected by mental symptoms, which was consistent with somatic symptoms. However, the characteristics of FC patients with somatic symptom remains unexplored.

**Methods:**

Clinical characteristics including somatic symptom (SOM, PHQ-15), depression (PHQ-9), anxiety (GAD-7), quality of life (PAC-QOL), constipation (KESS), demographic variables, anatomical abnormalities and symptoms were investigated. Subsequent analyses encompassed the comparison of clinical parameters between patients with SOM + group (PHQ-15 ≥ 10) and SOM- group (PHQ-15 < 10), subgroup analysis, correlation analysis, and logistic regression. Lastly, we evaluated the somatic symptom severity (SSS) among FC patients subjected to various stressors.

**Results:**

Notable disparities were observed between SOM + and SOM- groups in variety of physiological and psychological variables, including gender, stressful events, sleep disorders, reduced interest, GAD-7, PHQ-15, PHQ-9, PAC-QOL, anterior rectocele, KESS, and internal anal sphincter achalasia (IASA) (*P* < 0.05). Subgroup analysis affirmed consistent findings across mental symptoms. Correlation analyses revealed significant associations between SSS and KESS, anterior rectocele, GAD-7, PHQ-9, and PAC-QOL (*P* < 0.05). Logistic regression identified PHQ-9 (OR = 7.02, CI: 2.06–27.7, *P* = 0.003), GAD-7 (OR = 7.18, CI: 2.00–30.7, *P* = 0.004), and KESS (OR = 16.8, CI: 3.09–113, *P* = 0.002) as independent predictors of SSS. Elevated SSS scores were significantly associated with couple, parental, and work-related stressors (*P* < 0.05).

**Conclusion:**

A marked heterogeneity was observed between SOM + and SOM- patients of FC, with SOM + accompanied by more severe constipation, anxiety and depression symptoms. This finding underscores the importance of considering somatic symptoms in diagnosis and treatment of FC.

## Introduction

Functional constipation (FC) is a common gastrointestinal disorder that is frequently characterized by difficult or infrequent bowel movements, painful defecation, and/or the sense of incomplete evacuation of stool [1]. The global prevalence of functional constipation across all studies was 14% [2]. Risk factors of FC include women, older age, low socio-economic status, lack of physical exercise, etc. [3].

Constipation, which consists of varied symptoms, causes several physical and mental issues and has a significant influence on health and quality of life [4]. Through the brain-gut axis, mental diseases such as anxiety and depression also impair gastrointestinal function [5]. The symptoms of FC affected by mental symptoms was consistent with somatic symptoms. As an important predictor of gastrointestinal complaints, somatic symptom are also a serious mental symptom that is associated with a decline in quality of life, functional limitations, an increase in the use of medical services and absenteeism [6]. Previous research [7] revealed that the majority of irritable bowel syndrome (IBS) patients attending to tertiary care suffered somatic co-morbidities. In addition, the average somatization score of children with constipation was considerably higher than that of the control group [8]. However, the characteristics of FC patients with somatic symptom has not been investigated yet.

The objective of this study was to investigate the psychological characteristics of FC patients. The following are the precise aims of the study: (1) Analyze the clinical presentation differences between FC patients with high somatic symptom severity (SSS, SOM+) and SOM-, including demographic and clinical data, mental or psychological disorders, and constipation symptoms. (2) After adjusting for potential confounding factors, evaluate the risk factors related to SOM + among these psychological and disease-related variables.

## Methods

### Study design

This is a cross-sectional study conducted between July 2021 and July 2022. Totally 593 FC patients were recruited continuously from department of digestive surgery of Xijing Hospital affiliated to Fourth Military Medical University. FC was diagnosed according to the Rome IV criteria. General data including gender, age, education level, marriage, comorbidities, etc. was collected. Imaging examination was performed when clinically indicated to exclude any organic disease of the colon. Our study was approved by the Chinese Ethics Committee of Registering Clinical Trials (ChiECRCT20200151). Informed consent was initially obtained from all participants during the recruitment phase of the larger, ongoing prospective study (ChiCTR2000034379, 04/07/2020, https://www.chictr.org.cn/showproj.html?proj=54604). A large sample size was enrolled to minimize selection bias and increase the generalizability of our findings.

### Inclusion and exclusion criteria

Inclusion criteria included (1) ages between 18∼80 years; (2) confirmed diagnosis of FC through Rome IV criteria; (3) adequate reading and comprehension ability in Chinese; (4) patients who voluntarily signed informed consent before enrollment. Exclusion criteria included (1) pregnant or lactating women; (2) heart disease, organ failure, immune disease and infection; (3) co-morbid gastrointestinal organic diseases such as tuberculosis, polyps, Crohn’s disease, tumors, etc.; (4) history of abdominal surgery; (5) history of mental disorders or medication of psychotropic drugs; (6) irritable bowel syndrome with Constipation (IBS-C), hypothyroidism, and Parkinson’s disease.

### Questionnaires and interviews

To assess patients’ psychological distress, following questionnaires were adopted: Patient Health Questionnaire somatic symptom severity scale (PHQ-15) [9], Patient Health Questionnaire-depression scale (PHQ-9) [10], Generalized Anxiety Disorder 7-item (GAD-7) [11], Patient Assessment of Constipation Quality of Life (PAC-QOL) [12], Knowles Eccersley Scott Symptom Score (KESS) [13]. Cut-off values for PHQ-9 and GAD-7 were ≥ 10. Several other factors potentially affecting patients’ mental symptoms were judged by means of interviews, including previous treatment effect, presence of stress, sleep disorders, and reduced interest within last 1 year.

Five sources of stress were identified by referring to the 12th question of PHQ questionnaire combining with clinical practices: (1) Couple Stress: difficulties of husband/wife, partner/lover or boyfriend/girlfriend; (2) Parental Stress: pressure caused by taking care of children, parents or other family members; (3) Work Stress: pressure from work, outside home or at school; (4) Peer Stress: pressure caused by colleagues; (5) Bad Event: something bad that happened recently. Those stressors above were conducted by interview to assess the main source of stress in recent years.

### Clinical examinations

Functional and anatomical abnormalities were evaluated through defecography, and pathological diagnosis was defined as follows. Perineal descent was diagnosed by measuring the distance > 30 mm between the anorectal junction and the pubococcygeal line (PCL) [14]. Anterior rectocele was defined as > 30 mm outpouching of the anterior rectal wall. Rectal intussusception was defined as an invagination of the rectal wall, either intrarectal, intra-anal, or an external prolapse of the whole circumference [15]. Pelvic floor dyssynergia was defined as anorectal angle (ARA) widening < 10° and/or the opening of anal canal < 10 mm, and/or anal canal opening > 10 mm in more than 30 s or interrupted by repetitive squeezing contractions [16]. Pelvic floor hernias (PFH) were diagnosed and assessed by the expansion of enterocele, omentocele, and sigmoidocele extending below the PCL reference line with sagittal diameter > 2 cm [17]. Puborectalis muscle hypertrophy was recognized gby smaller ARA, longer anal canal, with contrast agent not or less expulsion. Distance between sacrum and rectum (DSR) was measured from posterior border of rectum to anterior border of sacrum with normal value < 10 mm.

The rectal pressure was then measured by anorectal manometry. Internal anal sphincter achalasia (IASA) was diagnosed by the absence of recto-sphincteric reflex on rectal balloon inflation and the presence of marked rhythmic activity. Colon transit time (CTT) was measured by the Metcalf technique. Normal upper limit for total colon transit time was set to ≤ 70 h for women and ≤ 60 h for men [18].

### Statistical analysis

R (4.2.1) was used for statistical analysis and plotting. Continuous variables are expressed as medians (interquartile ranges). Categorical variables are shown as the number and proportion. Patients with a high score of PHQ-15 were categorized into SOM+ (somatic symptom positive) group while those with low score of PHQ-15 were classified into SOM- (somatic symptom negative) group. The Venn diagram was generated by ggVennDiagram package in R. Correlations between SSS and clinical variables were evaluated using the Pearson correlation coefficient. Multivariate logistic regression was used to identify independent risk factors of SSS. Multicollinearity analysis was conducted to assess the relationships among the independent variables. χ² test or Fisher’s exact test was used to compare categorical variables between subgroups. Continuous variables were compared by the student’s *t* test or Mann Whitney *U* test. *P* < 0.05 was considered statistically significant. Missing data were handled by means of single imputation for missing completely at random data or multiple imputation procedure for missing at random data. STROBE statement was followed to prepare this study.

## Results

### Demographic characteristics

**Fig. 1:**
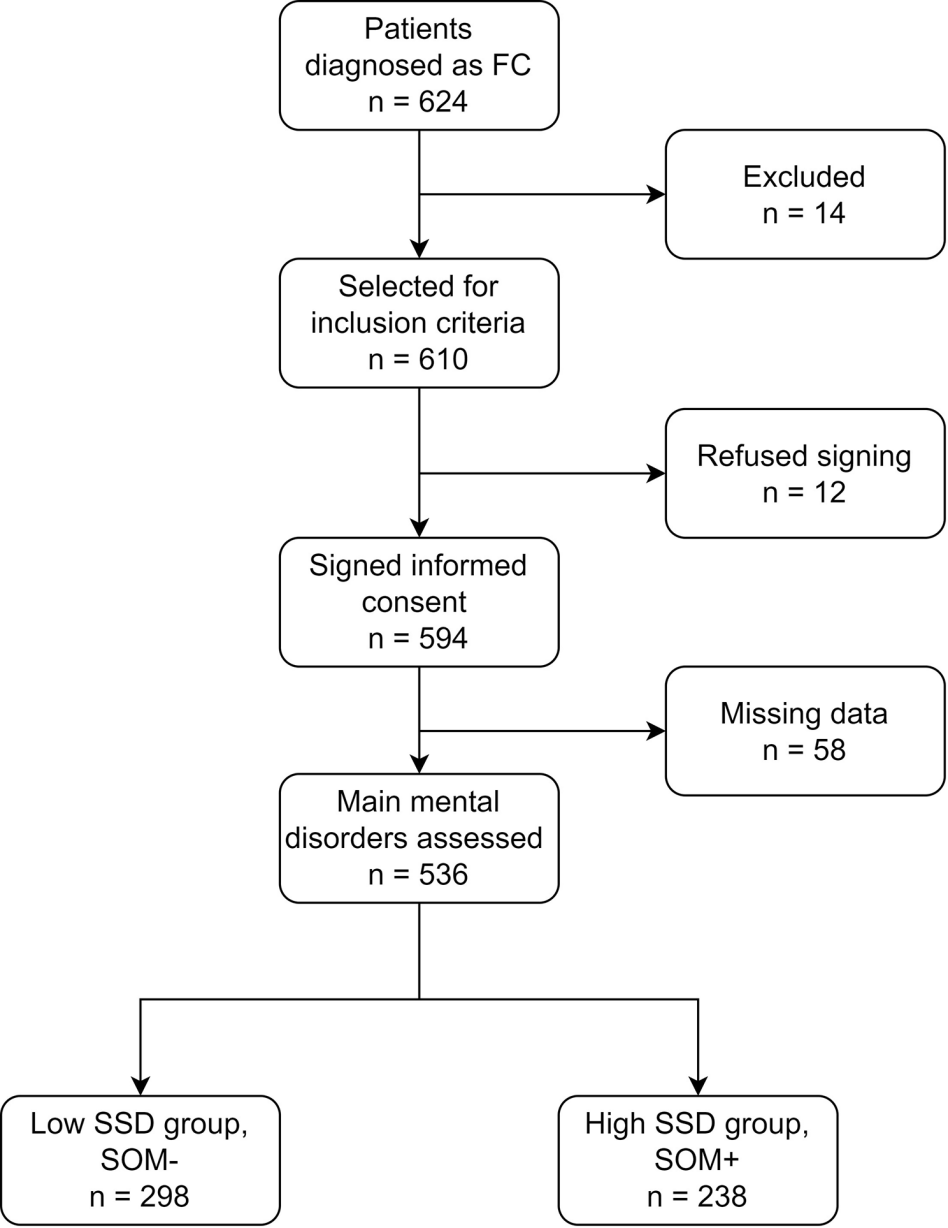
Flowchart of cohort integration

Our research involved 594 FC patients in total (Fig. 1). The median age of FC patients was 46, and 421 (70.9%) of them were female. The majority of patients (220, 77.5%) were married. Few individuals were diagnosed with hypertension (58, 9.9%) and diabetes (33, 5.9%). The number of SOM- and SOM + patients was comparable (298, 55.6% vs. 238, 44.5%). The median scores for PHQ-15, PHQ-9, PAC-QOL, and KESS were respectively 9, 7, 54, and 17. The majority of patients reported neither stress (489, 82.5%) nor stressful events (513, 86.8%) In addition, a substantial number of patients reported sleep disorders (277, 46.9%) and reduced interest (260, 44.0%). The previous treatment outcome of the majority of FC patients was unsatisfactory, with 186 ineffective instances (36.7%), 302 short-term effective cases (59.6%), and just 19 effective cases (3.3%). Perineal descent (81.9%), Rectal intussusception (81.1%), abnormal rectal pressure (57.6%), abnormal CTT (49.5%), anterior rectocele (48.8%), pelvic floor hernia (29.2%), IASA (9.4%), pelvic floor dyssynergia (5.4%) and puborectalis muscle hypertrophy (5.0%) were the most significant comorbid functional or anatomical abnormalities successively in FC patients. The results are shown in Table 1.

**Table 1:** Characteristics of functional constipation patients

Characteristic	*N* = 594
Age	46 (33, 59)
Gender	
Male	172 (29.0%)
Female	421 (71.0%)
Marriage	
Single	44 (15.5%)
Divorced	20 (7.0%)
Married	220 (77.5%)
Hypertension	
Yes	58 (9.9%)
No	527 (90.1%)
Diabetes Mellitus	
Yes	33 (5.6%)
No	552 (94.4%)
Education	
Elementary school	91 (16.9%)
Middle school	119 (22.1%)
Junior college	98 (18.2%)
High school	93 (17.3%)
University degree	138 (25.6%)
Somatic symptom	
SOM−	298 (55.6%)
SOM+	238 (44.4%)
PHQ-15	9 (5, 12)
PHQ-9	7 (3, 11)
GAD-7	6.0 (2.0, 11.0)
PAC-QOL	54 (38, 72)
KESS	17 (12, 21)
Stress	
Yes	104 (17.5%)
No	489 (82.5%)
Sleep disorders	
Yes	277 (46.9%)
No	314 (53.1%)
Reduced interest	
Yes	260 (44.0%)
No	331 (56.0%)
Treatment effect	
ineffective	186 (36.7%)
short-term effective	302 (59.6%)
effective	19 (3.7%)
Puborectalis hypertrophy	
Abnormal	7 (5.0%)
Normal	132 (95.0%)
IASA	
Abnormal	13 (9.4%)
Normal	126 (90.6%)
Rectal pressure	
Abnormal	87 (57.6%)
Normal	64 (42.4%)
CTT	
Abnormal	103 (49.5%)
Normal	105 (50.5%)
Intussusception	
Abnormal	116 (81.1%)
Normal	27 (18.9%)
Perineal descent	
Abnormal	122 (81.9%)
Normal	27 (18.1%)
Pelvic floor dyssynergia	
Abnormal	8 (5.4%)
Normal	139 (94.6%)
Pelvic floor hernia	
Abnormal	42 (29.2%)
Normal	102 (70.8%)
Anterior rectocele	
Abnormal	87 (48.9%)
Normal	91 (51.1%)

### Overlapping of depression, anxiety, and somatic symptom in FC patients

**Fig. 2:**
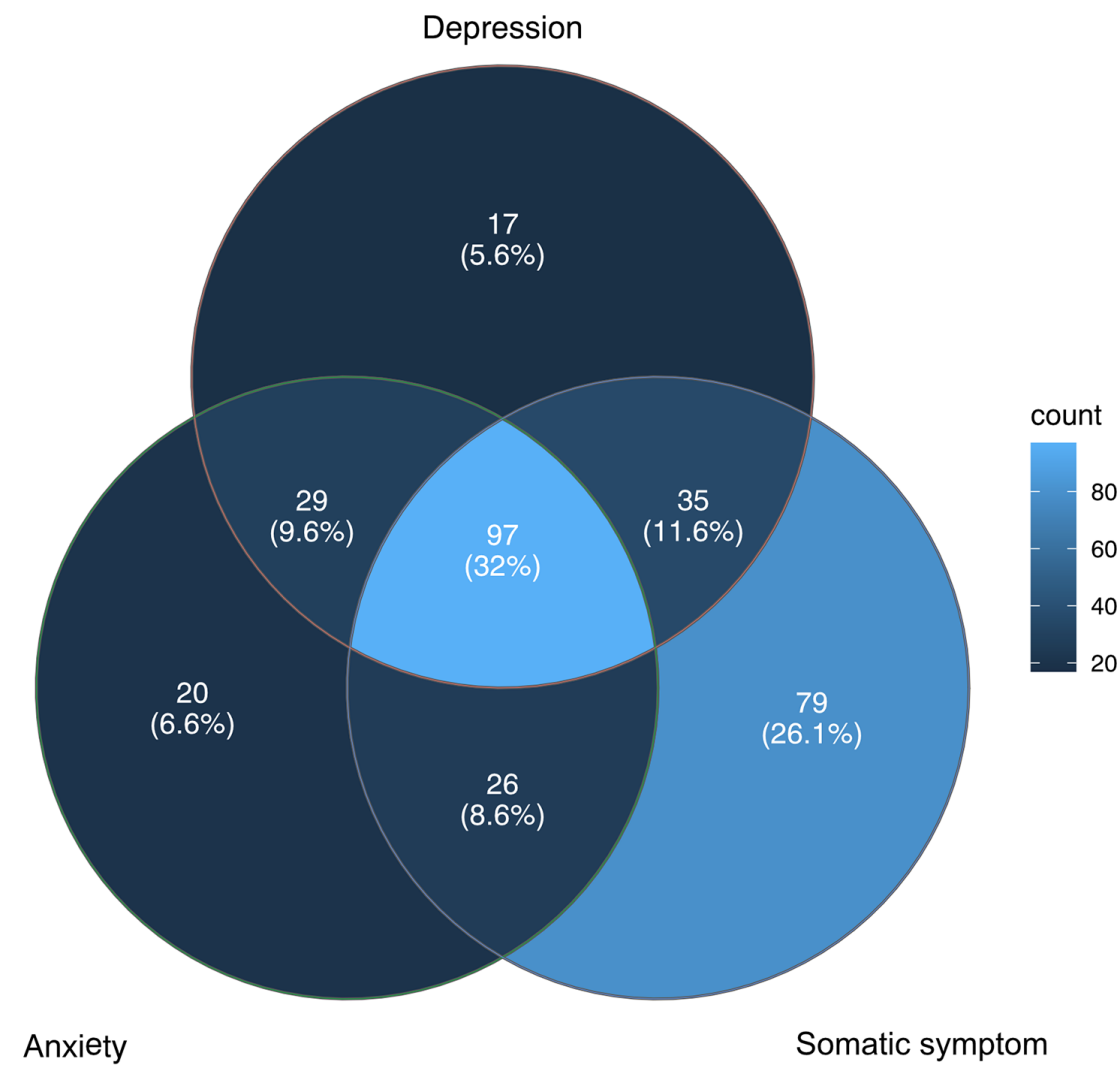
Overlapping of depression, anxiety, and somatic symptom in FC patient

A total of 303 patients (56.64%) out of 536 FC patients who completed the questionnaires reported at least one physical complaint. The prevalence of depression, anxiety, and somatic symptom were, respectively, 58.8%, 56.8%, and 78.3%. Among these patients, 97 (32.0%) were positive screened for all three mental disorders concurrently. While 17 (5.6%) were screened positive solely for depression, 20 (6.6%) were positive solely for anxiety, and 79 (26.1%) were positive only for somatic symptom. The results are shown in Fig. 2.

### Relationship between somatic symptom and clinical characteristics

Somatic symptom was classified into SOM + and SOM- based on PHQ-15 by cutoff value of 10, and the link between somatic symptom and other clinical factors was examined. First, differences in general clinical characteristics and stress-related parameters were evaluated between the SOM- and SOM + groups. The proportion of women, patients with stress events, less than a middle school education, sleep disorders, and reduced interest were substantially greater (*P* < 0.05) among SOM + patients. Similarly, the GAD-7, PHQ-15, PHQ-9 and PAC-QOL scores of SOM + patients were considerably greater than those of SOM- patients (*P* < 0.05). The study of parameters associated with constipation revealed that patients with SOM + had a higher KESS score, a more severe anterior rectocele, and a smaller proportion of IASA (*P* < 0.05). There was no significant difference between two groups in the distribution of other anatomical anomalies. The results are shown in Table 2.

**Table 2:** Relationship between clinical variables and somatic symptom (PHQ-15).

Characteristic	SOM-, *N* = 298	SOM+, *N* = 238	*P* value
Age	45.48 ± 16.82	47.87 ± 15.88	0.092
18∼30	80 (19%)	24 (23%)	
30∼45	127 (30%)	35 (33%)	
45∼60	130 (30%)	19 (18%)	
≥ 60	91 (21%)	27 (26%)	
Gender			0.021*
Male	96 (32%)	55 (23%)	
Female	202 (68%)	183 (77%)	
Marriage			0.678
Single	25 (17%)	19 (15%)	
Divorced	9 (6.0%)	11 (8.7%)	
Married	117 (77%)	96 (76%)	
Education			0.006*
Middle school and lower	99 (34%)	108 (46%)	
Higher than middle school	196 (66%)	129 (54%)	
Hypertension			0.887
Yes	30 (10%)	25 (11%)	
No	265 (90%)	210 (89%)	
Diabetes Mellitus			> 0.999
Yes	18 (6.1%)	14 (6.0%)	
No	277 (94%)	221 (94%)	
Stressful events			0.006*
Yes	31 (10%)	45 (19%)	
No	266 (90%)	192 (81%)	
Sleep disorders			< 0.001*
Yes	111 (37%)	137 (58%)	
No	186 (63%)	100 (42%)	
Reduced interest			< 0.001*
Yes	106 (36%)	127 (54%)	
No	191 (64%)	110 (46%)	
GAD-7	5.12 ± 4.89	9.86 ± 5.70	< 0.001*
PHQ-15	5.50 ± 2.41	13.03 ± 3.25	< 0.001*
PHQ-9	5.14 ± 4.75	11.65 ± 6.32	< 0.001*
PAC-QOL	63.18 ± 22.13	148.95 ± 20.98	< 0.001*
KESS	16.23 ± 5.50	19.08 ± 6.18	< 0.001*
DSR	3.85 ± 11.09	5.30 ± 13.07	0.522
Perineal descent	41.17 ± 20.75	40.50 ± 18.49	0.840
Pelvic floor dyssynergia	122.75 ± 32.59	121.51 ± 23.70	0.791
Pelvic floor hernia	7.22 ± 16.55	5.31 ± 12.32	0.434
Anterior rectocele	7.71 ± 10.16	13.29 ± 11.59	0.001*
Puborectalis hypertrophy			> 0.999
Abnormal	4 (4.6%)	2 (4.0%)	
Normal	83 (95%)	48 (96%)	
CTT			0.449
Abnormal	51 (48%)	39 (54%)	
Normal	55 (52%)	33 (46%)	
Rectal pressure			0.115
Abnormal	48 (54%)	36 (68%)	
Normal	41 (46%)	17 (32%)	
IASA			0.004*
Abnormal	12 (14%)	0 (0%)	
Normal	76 (86%)	49 (100%)	

### Subgroup analysis

**Fig. 3:**
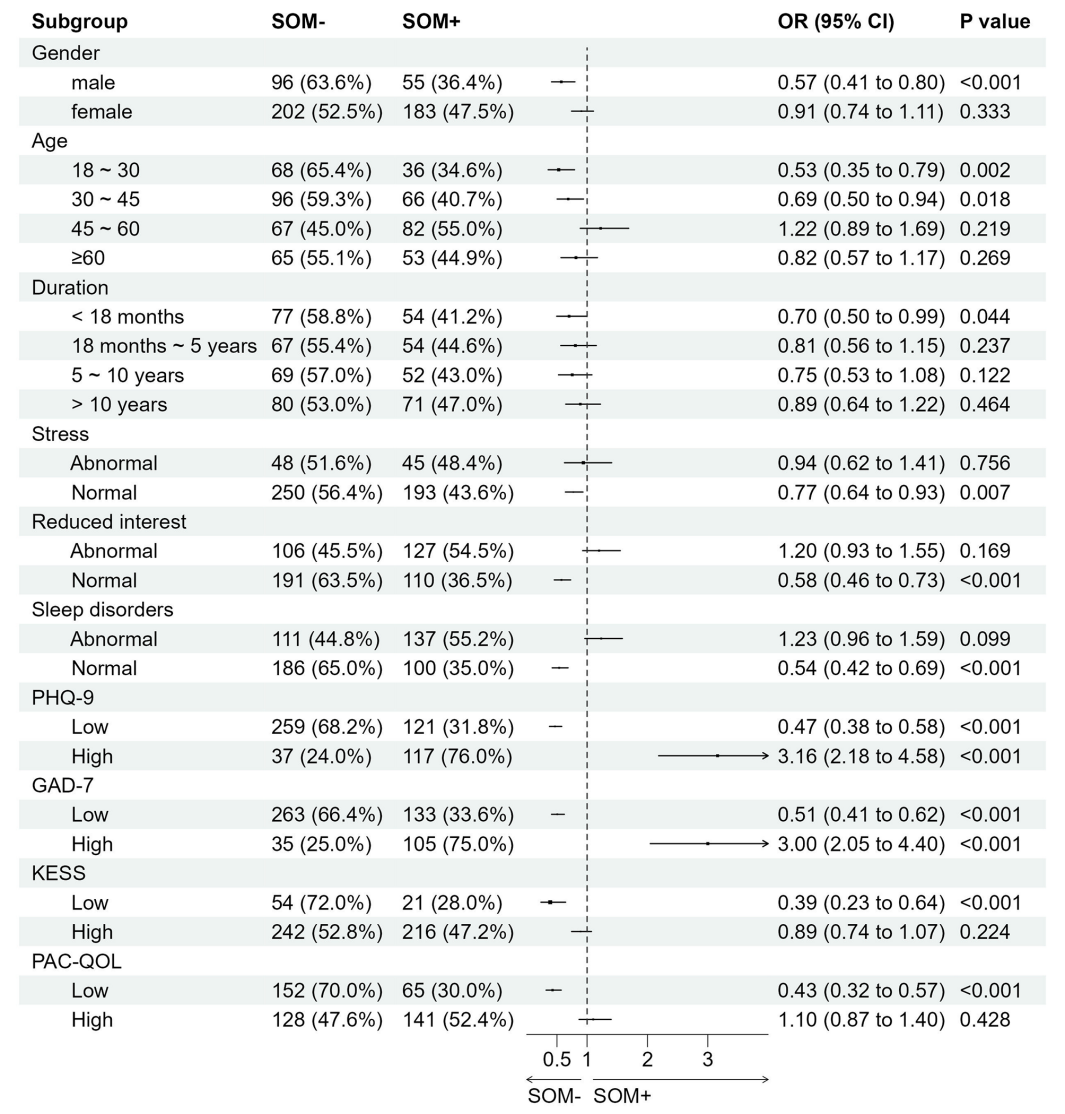
Subgroup analysis for SOM + and SOM- groups

Through our subgroup analysis, we examined the distribution of somatic symptom among diverse subgroups of FC (Fig. 3). SOM- had a higher proportion in subgroups below: male (*P* < 0.001), age of 18∼30 and 30∼45 (*P* < 0.05), duration less than 18 months (*P* = 0.044), without stress (*P* = 0.007), without reduced interest (*P* < 0.001), without sleep disorders (*P* < 0.001), lower KESS (*P* < 0.001) and PAC-QOL (*P* < 0.001) scores. Additionally, the prevalence of SOM- is notably elevated among patients with negative PHQ-9 and GAD-7 scores, as compared to those with positive scores (*P* < 0.001). Taken together, the evidence strongly indicated a significant association of SOM- with the majority of the subgroups analysed. Conversely, SOM + appears to be more closely related to mental symptoms.

### Correlations between somatic symptom and constipation related variables

The Spearman correlation analysis revealed substantial positive relationships (*P* < 0.05) between the somatic symptom (PHQ-15) and the KESS score, GAD-7, PHQ-9, and PAC-QOL. Among them, the correlation coefficient between PHQ-9 and PHQ-15 was 0.647, and variables having a correlation coefficient larger than 0.4 included GAD-7 (*r* = 0.498) and PAC-QOL (*r* = 0.403). The correlation coefficients of KESS and anterior rectocele were all less than 0.4. The results are shown in Table 3.

**Table 3:** Spearman correlation analysis between constipation related variables and somatic symptom (PHQ-15).

Characteristic	r	*P* Value
KESS	0.350	< 0.001*
DSR	0.091	0.303
Perineal descent	0.033	0.691
Age	0.056	0.197
Pelvic floor dyssynergia	-0.137	0.100
Pelvic floor hernia	-0.017	0.836
Anterior rectocele	0.182	0.017*
GAD-7	0.498	< 0.001*
PHQ-9	0.647	< 0.001*
PAC-QOL	0.403	< 0.001*

### Logistic regression analysis of somatic symptom in FC patients

The analysis for multicollinearity did not indicate any presence of significant multicollinearity among the variables. Adjusted for pertinent sociodemographic, physiological, and psychological variables, binary logistic regression analysis demonstrated a significant association between SOM + and depression (PHQ-9, OR = 7.02, *P* = 0.003), anxiety (GAD-7, OR = 7.18, *P* = 0.004), and constipation (KESS, OR = 16.8, *P* = 0.002). The results were shown in Table 4.

**Table 4:** Logistic regression analysis of SOM+ (PHQ-15 ≥ 10) in FC patients

Characteristic	OR	95% CI	*P* value
Anterior rectocele			
Normal	—	—	
Abnormal	0.95	0.25, 3.53	> 0.9
KESS			
Low	—	—	
High	16.8	3.09, 113	0.002*
PAC-QOL			
Low	—	—	
High	1.30	0.36, 4.46	0.7
GAD-7			
Low	—	—	
High	7.18	2.00, 30.7	0.004*
PHQ-9			
Low	—	—	
High	7.02	2.06, 27.7	0.003*
Gender			
female	—	—	
male	0.44	0.10, 1.86	0.3
Reduced interest			
No	—	—	
Yes	1.67	0.55, 5.28	0.4
Sleep disorders			
No	—	—	
Yes	1.19	0.39, 3.66	0.8

OR = Odds Ratio, CI = Confidence Interval

### Relationship between somatic symptom and psychosocial stressors

**Fig. 4:**
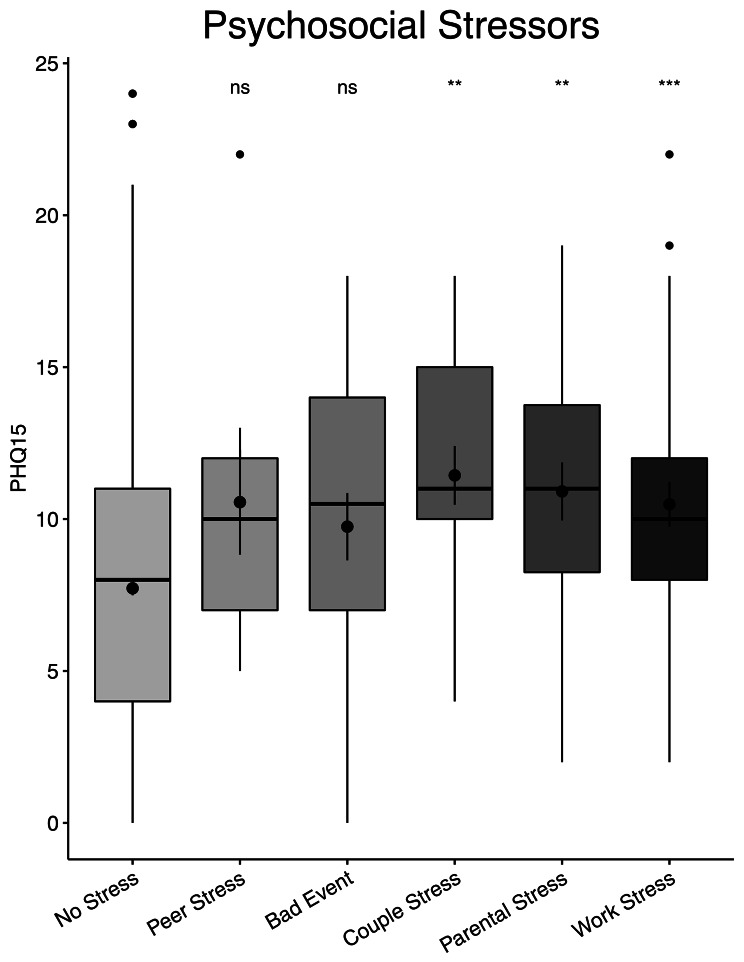
Odds of having SSD for patients exposed to different psychosocial stressors

The relationship between common psychosocial stressors and somatic symptom was studied, including peer stress, bad event, couple stress, parent stress, and work stress. Patients with couple stress, parent stress, and work stress had significantly higher PHQ-15 ratings than those without stress (*P* < 0.05, Fig. 4).

## Discussion

This research assessed the psychological features of FC patients. First, we explored the overall characteristics of FC patients, then investigated the distribution and overlapping of anxiety, depression, and somatic symptoms. Afterwards, clinical characteristics of SOM- and SOM + patients were compared was conducted. Next, a correlation analysis and logistic regression between clinical parameters and somatic symptom was carried out. Lastly, we examined the impact of exposure to various social and psychological stressors on somatic symptom. The results indicated that SOM + frequently corresponds to a more severe type of FC and is closely associated with constipation symptoms, anxiety, and depression.

Initially, the general condition of FC patients was examined. Similar to other study, we discovered that women were over twice as likely as males to have constipation. Numerous hypotheses have been proposed to explain this occurrence. For instance, variations in progesterone and estrogen levels decrease the intestinal migration of women [19], or the obstetric history of women causes harm to their pelvic floor [20]. Age is an additional factor that may influence FC. The incidence of constipation is higher in younger and middle-aged patients, according to research [21]. According to our cross-sectional study, the median age of FC patients is 46 years old, and 50% of patients are between the ages of 33 and 59, which is consistent with previous studies.

In addition, 46.9% of FC patients in our research had a combination of sleep disturbances, which was consistent with previous study [22]. Compared to healthy individuals, patients with functional constipation are more likely to suffer from depression and anxiety [23]. In accordance with previous research, our data also indicated that FC patients have higher PHQ-15, PHQ-9, and PAC-QOL levels.

Constipation is frequently accompanied by mental disorders such as anxiety, depression, and anorexia nervosa [24]. In a study comparing 47 women with idiopathic constipation versus 28 healthy controls, researchers found that the patients had much higher levels of depression and anxiety [25]. Adibi et al. [26] conducted more research on a broader scale. There was a total of 2,560 non-constipated patients and 802 constipated patients recruited, and it was discovered that the proportion of constipation patients with depression rose dramatically. Inversely, those with depression are also substantially more likely to suffer from constipation [5]. It indicates a complicated relationship between constipation and mental illnesses.

Anxiety, depression, and somatic symptoms have complex interactions [27]. Depression and anxiety are capable of eliciting physical symptoms [28]. In the opposite direction, somatic symptom also contribute to the development of anxiety and depression [29]. One of the probable etiologies is physical difficulty or limitation produced by somatic symptom. Furthermore, shared etiological variables such as environmental, psychological, and biological factors might all contribute to the incidence of depression, anxiety, and somatic symptom [30].

Nevertheless, anxiety, depression and somatic symptoms still differ from one another despite the association. According to Bekhuis et al. [31], depression and anxiety had significant and partly different associations with somatic symptom. In our study, 56.6% of FC patients had mental health issue. Among these patients, the incidences of depression, anxiety, and somatic symptom were 58.8%, 56.8% and 78.3%, respectively, with 6.6%, 5.8% and 26.1% for exclusive diagnosis. Somatic symptoms also constituted the primary complaints in FC patients frequently. While our study did not confirm causality between mental disorders and FC, it underscored the need for future research to elucidate the pathophysiological mechanisms. In summary, compared to anxiety and depression, somatic symptoms emerged more representative with a higher prevalence and lower comorbidity with other mental symptoms.

We finally confirmed the independent risk factors of somatic symptom including PHQ-9, GAD-7, and KESS. Firstly, we found that somatic symptom is associated with female, stressful events, sleep disorders, reduced interest, GAD-7, PHQ-15, PHQ-9, PAC-QOL, anterior rectocele, KESS, and IASA. According to the following correlation analysis, there is a substantial association between GAD-7, PHQ-9, and PAC-QOL with correlation coefficients more than 0.40. Furthermore, the logistic regression analysis identified PHQ-9, GAD-7, and KESS as independent risk factors of somatic symptom.

In summary, the results indicated a significant proportion of FC patients having aberrant somatic symptom, and these individuals are more likely to exhibit severe constipation symptoms and mental disorders. As mentioned previously, depression and anxiety were much higher in FC patients than in healthy individuals [4, 5]. Rajindrajith et al. [8] discovered that children with constipation had a wide range of somatic symptom. Additionally, Singh et al. [7] revealed that IBS patients had higher SSS than healthy people, and patients with higher SSS also tended to have more severe gastrointestinal symptoms. In our study, the characteristics of FC patients with somatic symptom was investigated. In conjunction with past research, we hypothesize that FC patients with somatic symptom may have a mutually promoting impact and deserves our attention.

Around 25–50% of somatic symptom are unexplained by organ pathology [32]. These symptoms might be caused by psychosocial factors [33]. We examined the association between common stressors and somatic symptom in this study. Those with couple stress, parental stress, and work stress scored considerably higher on the PHQ-15 than patients without stress. In fact, a significant proportion of constipation is induced by psychosocial factors that are mediated through the brain-gut axis, which includes neurological, neuroimmune, and neuroendocrine pathways [34]. For instance, the corticotropin-releasing factor (CRF) pathway linked with depression might result in disruption of the brain-gut axis, which increases the risk of constipation [35]. Somatic symptom were linked to adrenergic receptors and serotonin (5-HT) 4 transporters, which is a critical neurotransmitter in the brain-gut axis and plays a crucial role in intestinal motility [36]. This showed that 5-HT 4 might modulate the link between somatic symptom and FC. The precise brain circuit requires additional investigation.

### Limitations

This article has certain limitations. First, a large number of subjective feelings cannot be examined quantitatively, such as sleep disorders, stress, reduced interest, etc. These characteristics were mostly determined through interviews, which might lead to recall bias. Second, the PHQ-15 questionnaire only contained somatic symptom but lack psychological symptoms. Third, single-center research may result in selection bias and more severe symptoms. Lastly, being a cross-sectional study, this research can only propose correlations between variables but not causation.

## Conclusion

This study explored the correlation between FC and somatic symptom. FC individuals with SOM + and SOM- have clinical features that differ significantly. High SSS is correlated with more severe symptoms of constipation and mental symptoms. In addition, somatic symptoms emerged more representative with a higher prevalence and lower comorbidity compared with anxiety and depression. Future study should clarify the neurobiopsychosocial systems that underlie the function of somatic symptom in FC.

## Data Availability

The datasets generated and analyzed during the current study are not publicly available as the data are being used in next study but are available from the corresponding author on reasonable request.

## Abbreviations

CTT: Colon transit time
DSR: Distance between sacrum and rectum
PFH: Pelvic floor hernias
ARA: Anorectal angle
PCL: Pubococcygeal line
IBS: Irritable bowel syndrome
FGID: Functional gastrointestinal disorder
IASA: Internal anal sphincter achalasia
KESS: Knowles-Eccersley-Scott Symptom Questionnaire
PAC-QOL: Patient Assessment of Constipation Quality of Life
GAD: General Anxiety Disorder
PHQ: Patient Health Questionnaire
SSS: Somatic symptom severity
FC: Functional constipation
